# Supplementary material for: DhDIT2 Encodes a Debaryomyces hansenii Cytochrome P450 Involved in Benzo(a)pyrene Degradation—A Proposal for Mycoremediation
Source: J Fungi (Basel). 2022 Oct 30;8(11):1150. doi: 10.3390/jof8111150 (PMC9698926; doi:10.3390/jof8111150)
Supplement: Supplementary file 1 [file jof-08-01150-s001.zip › jof-1806656-supplementary.pdf]

Supplementary Figure S1.

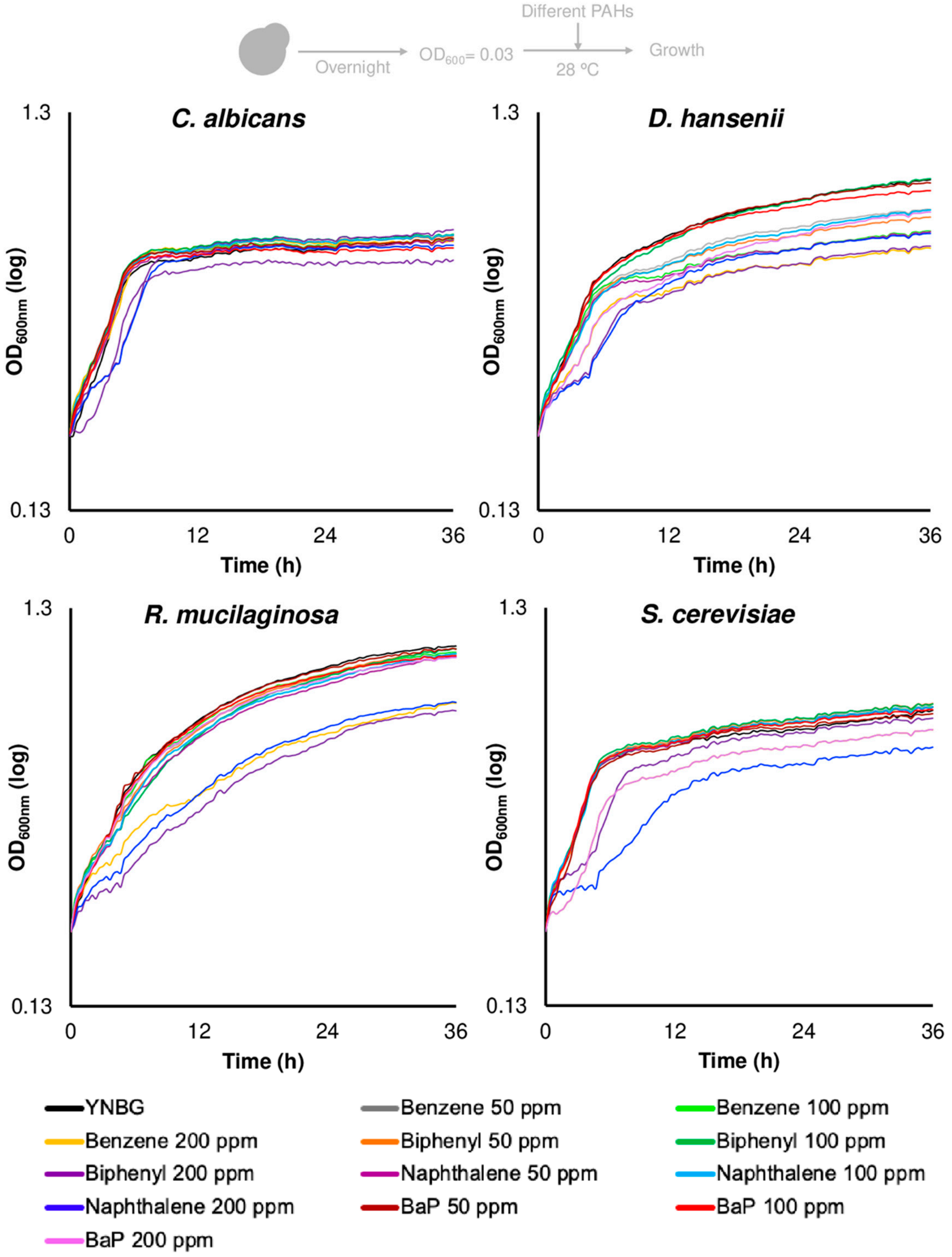

Figure S1. Effect of different PAHs on yeast growth. The effect of several concentrations (10 ppm to 500 ppm) of benzene, naphthalene, biphenyl, and BaP was studied on the growth of *C. albicans, D. hansenii, R mucilaginosa,* and *S. cerevisiae* at 28 °C. Cell density was measured every hour for 36 h in an automated BioscreenC plate reader. Each curve represents three biological replicates.

Supplementary Table S1. Genes encoding the yeast CYPs studied in this work

Yeast	Gene	Gene length (pb)	Exons	Aminoacids	Protein
C. albicans	*ALK1*	1581	1	526	CYP52A24
D. hansenii	*DEHA2C02596g (DhDIT2)*	1575	1	525	CYP52A44
S. cerevisiae	*DIT2*	1470	1	490	CYP56A1
R. mucilaginosa	*700567*	1815	8	605	CYP700567
	710473	1596	9	532	CYP710473
	680755	1626	10	542	CYP680755

To identify CYP homologues in *Candida albicans, Debaryomyces hansenii, Rhodotorula mucilaginosa,* and *Saccharomyces cerevisiae,* queries were performed in the following databases: Cytochrome P450 (https://drnelson.uthsc.edu/Cytochrome450.html) [29], Fungal cytochrome P450 database (http://p450.riceblast.snu.ac.kr/index.php?a=view) [30] ensemble fungi (https://fungi.ensembl.org/index.html) [31], and the *Saccharomyces* genome database website (https://www.yeastgenome.org) [32]. This research was conducted using the *AY515589* gene coding for the *Phanerochaete chrysosporium* CYP5144A7 protein and the *AN1884* gene coding for the *Aspergillus nidulans* CYP617D1 protein.

Supplementary Figure S2.

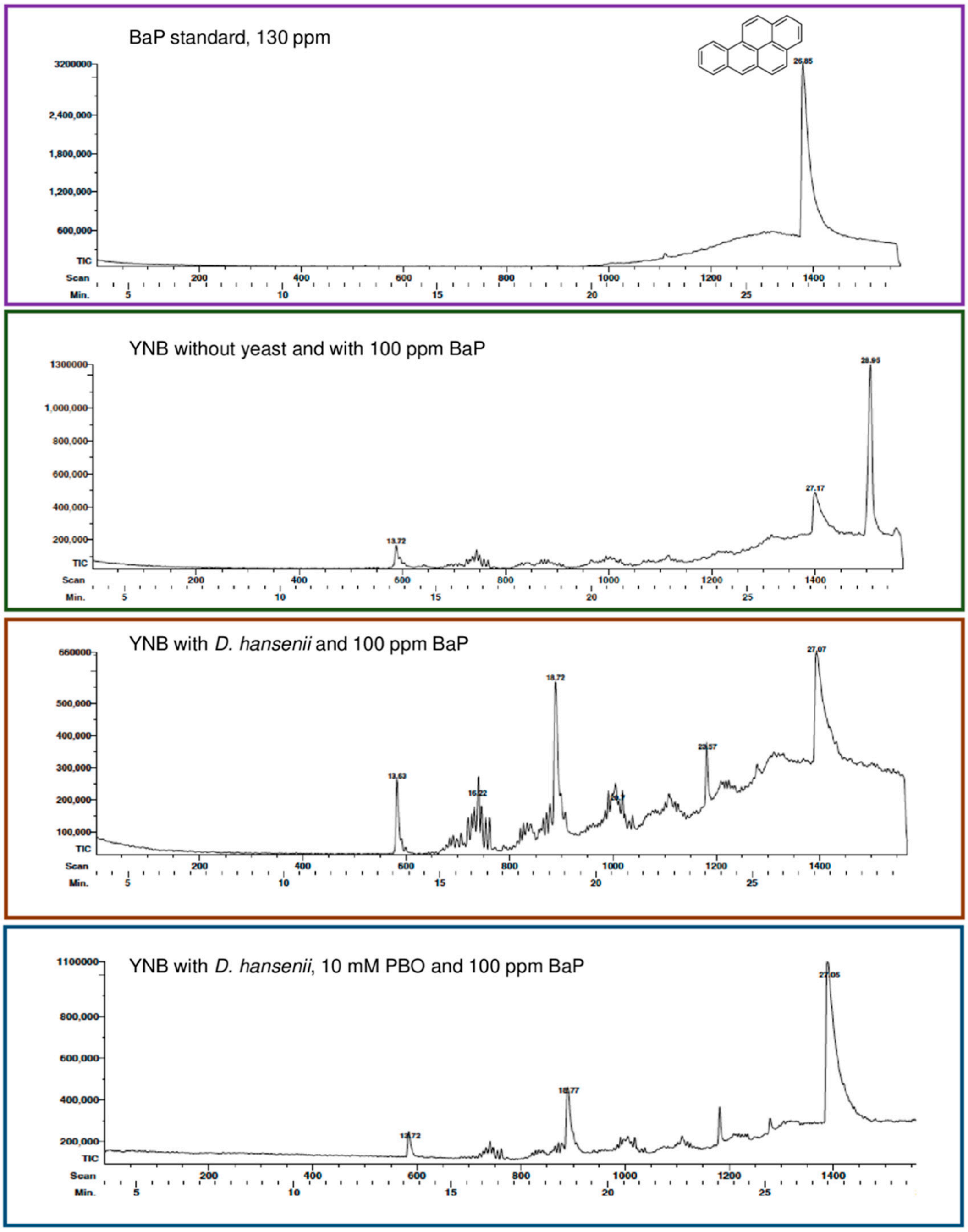

Figure S2. GC chromatograms for BaP degradation by *D. hansenii*. Chromatogram one was registered after analyzing a BaP standard (130 ppm, 500 μM). Chromatogram two: BaP extracted in a cell-free YNB culture medium containing BaP 100 ppm (396 μM) after 10 days. Chromatograms three and four: yeasts were grown for 24 h in YNBG medium and then transferred to a YNB supplemented containing 100 ppm BaP in the absence or presence of 10 mM PBO (CYP inhibitor), as indicated; after 10 days the non-metabolized BaP was extracted and analyzed. Representative chromatograms of the respective conditions rare shown.

Supplementary Figure S3.

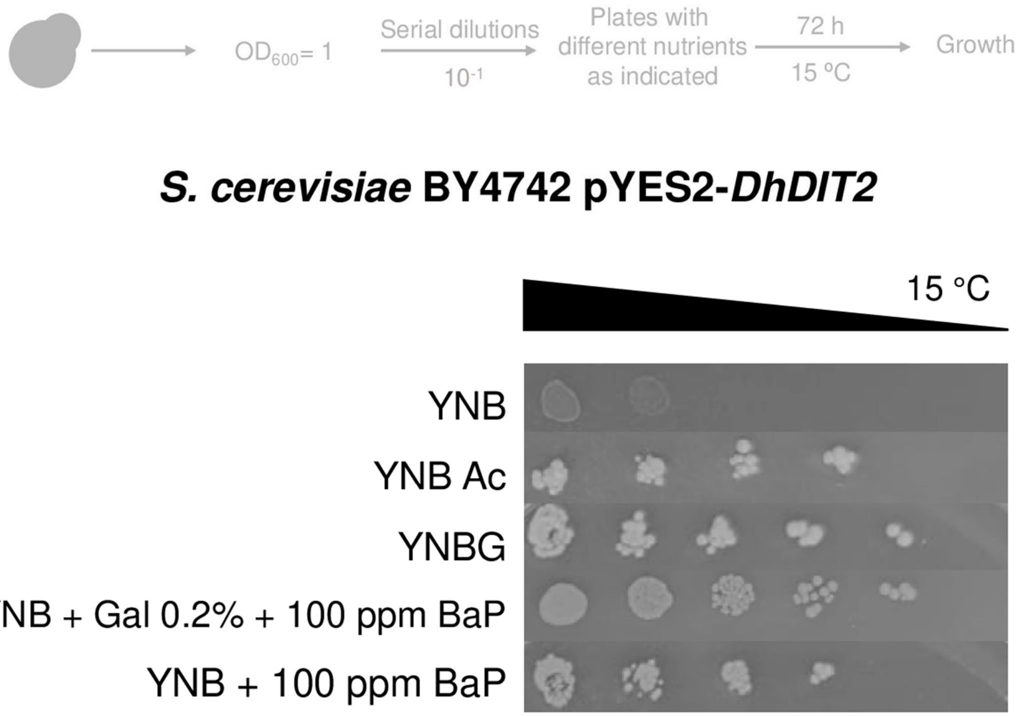

Figure S3. Effect of BaP on the growth of *S. cerevisiae* BY4742 pYES2-*DhDIT2*. Yeasts were grown overnight in a liquid YNBG medium and adjusted to OD_{600}= 1.0. Aliquots of 10-fold serial dilutions were spotted in the respective media and they were incubated at 15 °C for 72 h and photographed. A representative image of three independent experiments is shown.
